# Inflammation-associated depression in cholangiocarcinoma: impacts of surgical resection and radiotherapy

**DOI:** 10.1186/s13030-026-00350-6

**Published:** 2026-01-30

**Authors:** Tzu-Hsuan Sung, Yang-Chen Shen, Chia-Jui Yen, Yan-Shen Shan, Po See Chen

**Affiliations:** 1 Department of Psychiatry, National Cheng Kung University Hospital, College of Medicine, National Cheng Kung University, 138, Sheng-Li Road, Tainan, 70428 Taiwan; 2 Department of Psychiatry, Ditmanson Medical Foundation Chia-Yi Christian Hospital, Chia-Yi City, Taiwan; 3 Department of Oncology, National Cheng Kung University Hospital, College of Medicine, National Cheng Kung University, Tainan, Taiwan; 4 Department of Surgery, National Cheng Kung University Hospital, College of Medicine, National Cheng Kung University, Tainan, Taiwan; 5 Institute of Clinical Medicine, College of Medicine, National Cheng Kung University, Tainan, Taiwan; 6 Institute of Behavioral Medicine, College of Medicine, National Cheng Kung University, Tainan, Taiwan

**Keywords:** Cholangiocarcinoma, Depression, Inflammatory markers, Radiotherapy, Surgery

## Abstract

**Background:**

Cholangiocarcinoma (CCA) is an aggressive malignancy associated with systemic inflammation and a high psychological burden. However, the relationship between inflammation and depression in CCA remains poorly understood. This study aimed to investigate the association between systemic inflammatory markers and depression in patients with CCA.

**Methods:**

We conducted a prospective cohort study of patients aged ≥ 20 years with a diagnosis of cholangiocarcinoma treated at a single tertiary medical center between May 2021 and September 2024. Depression was evaluated using the Patient Health Questionnaire-9. Generalized linear mixed models were applied to examine the relationships between inflammatory markers and depression.

**Results:**

A total of 164 patients with CCA were included, comprising 97 intrahepatic cholangiocarcinoma (ICC) and 67 extrahepatic cholangiocarcinoma (ECC) cases. Depression prevalence was 19.9% at baseline and fluctuated over time, ultimately decreasing to 10.4% at 12 months. In multivariate analyses, not receiving surgical resection and higher C-reactive protein (CRP) levels were independently associated with depression in the overall CCA cohort and in the ICC subgroup. In the ECC subgroup, receipt of radiotherapy and lower albumin levels were independently associated with increased depression risk.

**Conclusions:**

Our study reveals a significant association between inflammatory markers and depression in CCA patients, with CRP emerging as a key marker. Notably, surgical intervention was associated with a reduced risk of depression, whereas radiotherapy in ECC patients was associated with an increased risk of depression.

**Supplementary Information:**

The online version contains supplementary material available at 10.1186/s13030-026-00350-6.

## Introduction

Cholangiocarcinoma (CCA) poses a significant medical challenge, as patients with this disease continue to have poor prognosis despite advancements in cancer therapies. CCA is anatomically classified as either intrahepatic cholangiocarcinoma (ICC) or extrahepatic cholangiocarcinoma (ECC) [1]. ICCs arise within the intrahepatic biliary tree as adenocarcinomas, which exhibit mass-forming, periductal infiltrative or intraductal growth patterns. The peak incidence for ICC occurs between ages 50 and 60, and the disease is especially prevalent in East Asia [2]. In contrast, ECC primarily affects the liver hilum epithelial cells, frequently presenting with obstructive jaundice and inflammatory complications [3].

The global incidence of CCA has risen in recent years, with mortality rates ranging from 1 to 2 per 100,000 in most regions to as high as 100 per 100,000 in endemic areas [4]. Late-stage diagnosis resulting from absence of early symptoms contributes to unfavorable treatment outcomes and substantial caregiver burden [3–5]. Liver and biliary tract cancers such as CCA rank as the third leading cause of cancer-related mortality worldwide, underscoring the urgent need for better disease understanding, therapeutic advancements, and improved palliative care and support systems [5, 6].

Recent clinical guidelines indicate that approximately 25% of cancer patients develop depressive disorders, markedly exceeding the 4% prevalence in the general population [7]. While some cancer populations demonstrate gradual reduction in depressive symptoms, phase-specific evidence in CCA remains scarce. Available longitudinal data suggest psychological symptoms in CCA patients remain relatively stable or show modest improvement during the early disease course [8]. Depression in CCA patients complicates disease management [9, 10] and correlates with reduced survival, decreased treatment adherence, and diminished quality of life [11].

Systemic inflammation has been implicated in both cancer progression and depression pathophysiology, prompting investigation of readily accessible inflammatory biomarkers in clinical settings. We focused on four markers routinely available through standard laboratory tests: C-reactive protein (CRP), an acute-phase inflammatory response indicator, neutrophil-to-lymphocyte ratio (NLR) reflecting innate and adaptive immune activation, platelet-to-lymphocyte ratio (PLR) suggesting inflammatory thrombocytosis, and albumin as a negative acute-phase reactant [12–14]. These markers derive from complete blood counts and metabolic panels, enabling clinical implementation without specialized handling or additional costs.

Previous studies demonstrate that CRP, NLR, PLR, and albumin reliably reflect systemic inflammation and are associated with CCA severity and prognosis [12, 13, 15]. CRP has also been linked to depression in cancer populations [14, 16]. However, no studies to date have specifically evaluated associations between these inflammatory markers and depression in patients with CCA, nor examined how different treatment modalities may influence these relationships.

To address these gaps, we conducted a longitudinal study to examine the course of depression prevalence over time in patients with CCA. We further examined whether inflammatory markers (CRP, NLR, PLR, and albumin) were associated with depression and whether surgical resection and radiotherapy influenced these associations. Given that surgical resection may alter systemic inflammation, we also evaluated treatment-related inflammatory profiles and explored potential ICC–ECC subtype differences. Identifying such indicators may enable earlier recognition of patients at higher risk of depression and facilitate timely psychiatric intervention to improve clinical outcomes.

## Methods

### Demographic and clinical characteristics

Demographic and clinical data for the patients were sourced from electronic medical records at National Cheng Kung University Hospital (NCKUH). These data included age, sex, cancer stage, and details regarding whether the patients underwent surgical resection, chemotherapy and radiation therapy. The study was a prospective, repeated-measures design with data collection points at baseline (before treatment) and at 2, 3, 4, 6, 9, and 12 months (during treatment). Inclusion criteria were: (i) age ≥ 20 years and (ii) a diagnosis of cholangiocarcinoma (stages I–IV) according to ICD-10 codes. Exclusion criteria included inability to provide informed consent or lack of essential clinical information required for diagnostic confirmation. Study participants were prospectively enrolled at NCKUH between May 2021 and September 2024. The study was approved by the Institutional Review Board of NCKUH (B-ER-110-060). All patients provided written informed consent prior to enrollment.

### Patient health Questionnaire-9 (PHQ-9)

Depression severity and screening were assessed using the PHQ-9, a widely recognized self-report tool consisting of nine questions. Each question offers four responses: 0 for “not at all,” 1 for “several days,” 2 for “more than half the days,” and 3 for “nearly every day.” Cumulative scores range from 0 to 27, and different ranges indicate varying levels of depression: 0–4 for minimal depression, 5–9 for mild depression, 10–14 for moderate depression, 15–19 for moderately severe depression, and 20–27 for severe depression [17].

​In this study, a PHQ-9 score of 10 or above was used as a threshold for significant depressive symptoms. ​This threshold is supported by existing literature and has proven reliability across diverse populations, particularly in oncology settings. The cutoff of 10 has a sensitivity of 0.77 and specificity of 0.94, making it an effective screening tool for depression in cancer patients [18, 19]. This operational definition enables precise identification of patients experiencing clinically significant depression, thereby expediting targeted psychological interventions.

### Inflammatory markers

Laboratory data for this study were extracted from the electronic medical records of NCKUH. The data encompassed measurements of a range of biomarkers such as CRP, NLR, PLR, and albumin levels. CRP level is a common marker for inflammation that is typically considered normal when below 10 mg/L, although this cutoff can vary slightly between laboratories. CRP levels at or above 10 mg/L are indicative of inflammation and serve as a crucial diagnostic marker in various clinical settings [20]. Albumin levels generally range from 3.5 to 5 g/dL, with lower levels (hypoalbuminemia) often associated with inflammatory states or chronic diseases [21].

To analyze potential multicollinearity among inflammatory markers, variance inflation factor (VIF) values were calculated. VIF values are commonly used to assess the degree of multicollinearity; values above 10 are typically considered an indication of significant multicollinearity that could affect regression analyses. In this study, the VIF values were found to be 2.07 for albumin, 1.97 for log-transformed CRP (Ln CRP), 1.44 for NLR, and 1.18 for PLR. ​Thus, multicollinearity is not likely to be a major concern for this dataset. All of the calculated VIF values are well below the threshold that indicates problematic multicollinearity, suggesting the statistical analyses are reliable.

### Statistical analysis

Descriptive statistics were applied to describe the demographic and clinical characteristics of the patient population. Continuous variables are presented as mean and standard deviation (SD), while categorical variables are shown as frequencies or percentages. The prevalence of depression (PHQ-9 score ≥ 10) among patients with CCA was expressed as percentage.

Continuous variables included age and inflammatory markers (set of four markers). Due to the non-normal distribution of CRP levels, the values were log-transformed using the natural logarithm (log e) for analysis. Initially, all variables were examined in the univariate analysis. Categorical variables were coded numerically for analysis. Male was coded as 1 and female as 0; female was taken as the reference. Cancer stage IV was coded as 1, while stages I, II, and III were coded as 0; the stage I-III group served as the reference. Surgical resection, chemotherapy and radiation therapy were coded similarly, with imputation applied to missing values as needed.

A generalized linear mixed model (GLMM) was used to analyze the relationships between inflammatory markers (CRP, NLR, PLR and albumin) and depression, while accounting for demographic and clinical variables. Variables showing statistical significance in the univariate analysis were included in a subsequent multivariate analysis. A significance threshold was set at a P-value of less than 0.05 for all statistical tests. Two-tailed P-values are reported. All statistical analyses were conducted using SPSS version 17 software (SPSS Inc., Chicago, IL) on Windows.

## Results

### Cohort demographics and prevalence of depression

This study enrolled 164 CCA patients (97 ICC, 67 ECC) treated between May 2021 and September 2024. The cohort consisted primarily of elderly men with advanced-stage disease (Table 1). Systemic chemotherapy was the primary treatment modality, while surgical resection and radiotherapy were performed less frequently. Among the 164 enrolled patients, 80 (48.8%) completed the 12-month follow-up assessment. Table S1 presents the retention status and dropout reasons at each time point throughout the study. In addition, baseline characteristics were compared between patients who completed the PHQ-9 and those who did not; the results are summarized in Table S2.

**Table 1:** Demographic and clinical characteristics of the patient cohort

	CCA (*N* = 164)		ICC (*n* = 97)		ECC (*n* = 67)	
Variable	N	%	N	%	N	%
Age^a^	65.58	± 11.65	63.11	± 11.85	69.14	± 10.46
Sex						
Female	71	43.3	44	45.4	27	40.3
Male	93	56.7	53	54.6	40	59.7
Tumor Stage						
I	24	14.6	15	15.5	9	13.4
II	25	15.2	4	4.1	21	31.3
III	23	14	13	13.4	10	14.9
IV	77	47	60	61.9	17	25.4
missing	15	9.1	5	5.2	10	14.9
Surgery						
Yes	30	18.3	18	18.6	12	17.9
No	134	81.7	79	81.4	55	82.1
Chemotherapy						
Yes	135	82.3	85	87.6	50	74.6
No	29	17.7	12	12.4	17	25.4
Radiation therapy						
Yes	15	9.1	10	10.3	5	7.5
No	149	90.9	87	89.7	62	92.5

^a^: Data are presented as mean ± SD

CCA, cholangiocarcinoma; ICC, intrahepatic cholangiocarcinoma; ECC, extrahepatic cholangiocarcinoma

Depression prevalence declined from 19.9% at baseline to 10.4% at the 12-month follow-up (Table 2). ECC patients exhibited higher baseline depression rates than ICC patients. Inflammatory markers (CRP, NLR, PLR) varied over time, paralleling changes in depression rates. In contrast, albumin levels remained relatively stable throughout follow-up.

**Table 2:** Descriptive statistics for inflammatory markers and depression (PHQ-9) scores

	Total *N*	Albumin				Natural log CRP				NLR			PLR			PHQ-9			
		*N*	Mean	SD	< 3.5, %	*N*	Mean	SD	≥ 10, %	*N*	Mean	SD	*N*	Mean	SD	*N*	Mean	SD	≥ 10, %
**CCA**																			
Month 0	164	119	3.65	0.75	34.5	105	43.17	56.67	65.7	124	7.67	16.33	124	234.94	205.35	84	6.11	5.37	19.9
Month 2	152	98	3.55	0.75	40.8	49	76.75	71.62	87.8	139	4.24	3.54	139	178.93	119.02	79	4.46	4.09	10.1
Month 3	146	97	3.73	0.74	26.8	55	37.88	67.72	45.5	129	4.47	5.13	129	183.30	138.93	64	5.36	5.72	20.3
Month 4	134	95	3.77	0.65	31.6	66	44.52	68.75	56.1	117	5.72	15.33	117	222.96	312.89	83	3.95	3.75	10.8
Month 6	127	97	3.77	0.73	28.9	77	33.99	51.03	46.8	107	5.82	10.81	107	183.92	164.01	78	4.68	4.48	11.5
Month 9	105	83	3.78	0.77	30.1	61	37.04	58.20	52.5	93	4.87	7.13	93	182.55	140.88	58	4.19	4.05	10.3
Month 12	80	65	3.91	0.63	29.2	56	35.58	60.93	46.4	68	3.84	4.04	68	179.56	144.68	48	3.92	4.68	10.4
**ICC**																			
Month 0	97	70	3.74	0.78	28.6	64	43.87	59.28	64.1	70	6.46	6.61	70	208.33	108.38	52	5.94	5.13	17.3
Month 2	90	53	3.62	0.75	35.8	27	78.89	68.06	88.9	81	4.24	3.07	81	173.33	102.48	52	4.58	4.09	11.5
Month 3	86	59	3.82	0.72	23.7	35	28.19	66.20	45.7	77	4.42	3.84	77	175.35	115.12	38	6.16	6.28	26.3
Month 4	80	58	3.83	0.68	25.9	40	47.95	78.94	55.0	73	6.22	18.75	73	221.42	358.77	50	3.50	3.27	6.0
Month 6	75	57	3.83	0.78	24.6	45	34.61	55.63	40.0	64	5.30	6.82	64	181.71	187.68	45	4.53	3.95	11.1
Month 9	61	49	3.95	0.65	20.4	38	25.79	48.41	44.7	55	3.68	3.71	55	168.63	136.72	39	4.13	4.51	12.8
Month 12	49	39	3.96	0.61	23.1	34	26.41	52.52	44.1	42	3.87	4.08	42	174.49	118.63	31	3.97	5.23	9.7
**ECC**																			
Month 0	67	49	3.51	0.68	42.9	41	42.07	53.04	68.3	54	9.23	23.61	54	269.44	283.60	32	6.38	5.81	21.9
Month 2	62	45	3.48	0.76	46.7	22	74.12	77.30	86.4	58	4.23	4.13	58	186.76	139.45	27	4.22	4.16	7.4
Month 3	60	38	3.58	0.76	31.6	20	54.85	68.66	45.0	52	4.55	6.64	52	195.07	168.70	26	4.19	4.67	11.5
Month 4	54	37	3.67	0.58	40.5	26	39.25	50.21	57.7	44	4.90	6.61	44	225.52	220.40	33	4.64	4.36	18.2
Month 6	52	40	3.68	0.64	35.0	32	33.12	44.61	56.3	43	6.60	14.96	43	187.22	122.63	33	4.88	5.18	12.1
Month 9	44	34	3.53	0.87	44.1	23	55.62	68.68	65.2	38	6.60	10.06	38	202.69	146.18	19	4.32	2.96	5.3
Month 12	31	26	3.82	0.68	38.5	22	49.76	71.00	50.0	26	3.78	4.05	26	187.76	181.39	17	3.82	3.59	11.8

Total N represents the number of participants who reached each time-point. Albumin was measured in grams per deciliter (g/dL). CRP was measured in units of mg/L

PHQ-9, Patient Health Questionnaire-9; CRP, C-reactive protein; NLR, neutrophil-to-lymphocyte ratio; PLR, platelet-to-lymphocyte ratio; CCA, cholangiocarcinoma; ICC, intrahepatic cholangiocarcinoma; ECC, extrahepatic cholangiocarcinoma

In ICC patients, inflammatory markers and depression rates showed greater variation over time than in ECC patients, representing descriptive trends rather than statistically tested subgroup differences (Table 2).

## Associations between depression and demographic or clinical variables

Multivariate GLMM analysis showed that higher Ln CRP levels and not receiving surgical resection were independently associated with depression in the overall CCA cohort (Table 3).

**Table 3:** Fixed effects on GLMM to identify factors associated with clinically significant depression (PHQ-9 ≥ 10)

	CCA (*N* = 164)				ICC (*n* = 97)				ECC (*n* = 67)			
Variable	Univariate Analysis		Multivariate Analysis		Univariate Analysis		Multivariate Analysis		Univariate Analysis		Multivariate Analysis	
	OR	*P*	OR	*P*	OR	*P*	OR	*P*	OR	*P*	OR	*P*
Age	1.009	0.574			1.017	0.442			0.993	0.801		
Sex (Male)	0.974	0.938			1.429	0.397			0.506	0.242		
Tumor Stage (Stage IV)	1.622	0.178			2.278	0.047	1.361	0.432	1.087	0.893		
Received surgery	0.217	0.004	0.332	0.022	0.242	0.021	0.31	< 0.001	0.169	0.1		
Received chemotherapy	2.089	0.292			--	--			1.396	0.671		
Received radiation therapy	1.403	0.407			0.731	0.575			4.019	0.002	6.329	0.005
Albumin	0.318	< 0.001	0.632	0.203	0.327	< 0.001	1.177	0.723	0.26	0.001	0.194	0.001
Natural log CRP	1.948	< 0.001	1.501	0.02	2.176	< 0.001	2.051	0.001	1.626	0.023	0.952	0.849
NLR	1.076	0.02	1.011	0.714	1.163	0.001	1.008	0.903	1.031	0.308		
PLR	1.001	0.084			1.001	0.25			1.001	0.175		

Data were analyzed using a binomial logistic GLMM with a logit link function, considering each subject as a random effect variable

GLMM, generalized linear mixed model; PHQ-9, Patient Health Questionnaire-9; CCA, cholangiocarcinoma; ICC, intrahepatic cholangiocarcinoma; ECC, extrahepatic cholangiocarcinoma; CRP, C-reactive protein; NLR, neutrophil-to-lymphocyte ratio; PLR, platelet-to-lymphocyte ratio

In the ICC subgroup, higher Ln CRP levels and not receiving surgical resection remained significantly associated with depression in multivariate analysis (Table 3). In the ECC subgroup, receipt of radiotherapy was positively associated with depression, whereas lower albumin levels were independently associated with an increased risk of depression (Table 3).

## Associations between treatments and inflammatory markers in CCA patients

Surgical patients demonstrated significantly more favorable inflammatory profiles than non-surgical patients, characterized by elevated albumin and reduced inflammatory markers. These patterns remained consistent across both CCA subtypes (Table 4).

**Table 4:** Comparison of inflammatory markers across treatment groups (surgery vs. non-surgery) of CCA patients (*N* = 164)

Variable	Surgery	Mean	SD	*P*
Albumin	Yes	4.06	0.57	< 0.001
	No	3.64	0.74	
Natural log CRP	Yes	2.15	1.26	< 0.001
	No	3.02	1.38	
NLR	Yes	3.29	3.86	< 0.001
	No	5.87	11.56	
PLR	Yes	159.17	126.78	< 0.001
	No	206.75	200.64	

CCA, cholangiocarcinoma; CRP, C-reactive protein; NLR, neutrophil-to-lymphocyte ratio; PLR, platelet -to-lymphocyte ratio

## Discussion

Depression prevalence in our CCA cohort was 19.9% at baseline and declined to approximately 10% during follow-up. Depression prevalence peaked at diagnosis and gradually improved over time, which is consistent with observations in other cancer populations [22, 23]. A previous study reported consistently mild and stable depression rates among both radically treated and palliative CCA patients, despite treatment differences [8]. In contrast, our cohort included a substantially higher proportion of non-surgical cases (81.7%) and nearly half of the patients with advanced disease, potentially contributing to distinct symptom trajectories. These observations highlight the importance of early psychological screening at diagnosis, followed by longitudinal monitoring to capture the evolution of depressive symptoms over time.

Higher CRP levels emerged as the only inflammatory marker independently associated with depression in our analyses. This result is consistent with oncology studies demonstrating reproducible associations between CRP and depressive symptoms [14]. By comparison, NLR and PLR are influenced by physiological factors such as age, hydration status, and stress, which may reduce their specificity for depression in multivariate analyses [24].

Several biological mechanisms underlie the association between CRP and depression. CRP elevation reflects interleukin-1 (IL-1), interleukin-1 (IL-6), and tumor necrosis factor-α (TNF-α) signaling, which alters blood–brain barrier permeability, activates microglia, and promotes neuroinflammatory changes contributing to depressive symptoms [16]. Elevated CRP has been associated with activation of the kynurenine pathway via induction of indoleamine 2,3-dioxygenase, reducing serotonin availability in ways implicated in depression [25]. These mechanisms explain the observed association between systemic inflammation and depressive symptoms across medical conditions [16, 26].

Although NLR and PLR showed univariate associations with depression, these ratios represent broad indicators of immune dysregulation. Elevations in NLR and PLR reflect peripheral inflammatory activation, which may trigger neuroimmune pathways and contribute to mood symptoms [16, 24]. However, their limited specificity may explain why their associations did not persist after adjustment for CRP.

Lower albumin levels were associated with an increased risk of depression, particularly among ECC patients. Hypoalbuminemia reflects systemic inflammation, nutritional deficiency, and greater chronic disease burden [27]. Low albumin is associated with reduced antioxidant capacity and impaired neurotransmitter synthesis, as albumin functions as a tryptophan carrier for serotonin production [27–29]. Chronic inflammation reduces albumin synthesis and promotes oxidative degradation, reducing antioxidant capacity and altering tryptophan availability, which has been associated with depressive symptoms [27, 28].

Cytokine signaling involving IL-1, IL-6, and TNF-α induces cyclooxygenase-2 (COX-2) expression, which promotes prostaglandin synthesis and amplifies inflammatory activity in CCA [30, 31]. Prostaglandin-dependent pathways contribute to central neuroinflammatory responses through microglial activation and altered neurotransmitter regulation, mechanisms repeatedly implicated in inflammation-related depression in experimental and translational studies [16, 32]. Clinical data further support this relationship, showing that cancer patients with elevated CRP experience more severe depressive symptoms [33]. Together, these findings suggest that CRP may serve as a clinically meaningful indicator for identifying patients at elevated psychological risk.

Surgical intervention was associated with reduced depression risk. Surgical candidates demonstrated higher albumin and lower inflammatory markers, reflecting better baseline physiological reserve. Elevated preoperative CRP is strongly associated with postoperative complications and mortality [34], and high CRP predicts poorer CCA outcomes [35]. Therefore, non-surgical patients in our cohort likely carried a higher inflammatory burden that contributed both to surgical ineligibility and to increased vulnerability to depression.

Patterns observed across other cancers further support these interpretations. In pancreatic cancer, patients with pre-existing depression are less likely to undergo surgery and have poorer postoperative survival [36], and early multimodal treatment reduces depression risk [37]. Our CCA findings parallel these observations. Breast reconstruction alleviates depressive symptoms [38], whereas lung cancer surgery has shown bidirectional associations with depression [39]. These examples emphasize the complex interactions between surgical treatment, systemic inflammation, and psychological outcomes. Surgical resection, when clinically appropriate, confers psychological benefits beyond oncological outcomes. Conversely, patients who remain non-surgical because of systemic inflammation or advanced disease may therefore require more intensive psychosocial support and closer monitoring.

Radiotherapy was independently associated with a higher risk of depression in ECC patients. This relationship likely reflects treatment-related symptom distress, psychological burden, and underlying disease severity. Radiotherapy generates sustained inflammatory responses through repeated release of reactive oxygen species, cytokines, and growth factors [40–42], mechanisms also implicated in depressive symptomatology. These inflammatory processes further suppress albumin synthesis, particularly in patients already susceptible to malnutrition due to biliary obstruction or cholestasis [27–29, 43].

Although systemic inflammation contributes to the association between albumin and depression in ECC patients, albumin and CRP reflect distinct biological processes. Albumin levels are shaped by nutritional status, biliary obstruction, and cholestasis—factors particularly relevant in ECC—and show stronger associations with psychological distress. CRP demonstrated limited variation within the ECC subgroup and showed reduced sensitivity to depression-related differences after covariate adjustment. These distinctions explain why low albumin, rather than CRP, emerged as an independent depression predictor in ECC patients.

These findings emphasize the need to address hypoalbuminemia and treatment-related inflammation in ECC patients. Nutritional support and inflammation-mitigating strategies, such as selective COX-2 inhibitors or corticosteroid-based regimens, could reduce both physical complications and psychological distress [31, 33, 44].

Overall, our results support a comprehensive approach to CCA care that integrates psychological assessment, inflammatory marker monitoring, and nutritional support. Early screening at diagnosis, routine monitoring of CRP to identify patients at increased risk, and proactive nutritional and psychosocial interventions, particularly for patients receiving radiotherapy, may improve quality of life and treatment outcomes.

Several limitations warrant consideration. First, although the Hospital Anxiety and Depression Scale (HADS) is designed to reduce somatic symptom impact, we employed the PHQ-9 because of its strong validation in cancer populations and routine clinical use. Certain PHQ-9 items overlap with cancer- or treatment-related physical symptoms, potentially introducing measurement bias. Physical complications and cancer-related somatic symptoms contribute to CRP elevation and partially overlap with depressive manifestations. Although multivariate models adjusted for major clinical covariates, residual confounding cannot be excluded. Nonetheless, the persistent association between CRP and depression after adjustment suggests CRP reflects underlying cytokine-mediated inflammatory processes rather than merely capturing physical symptom burden. A further limitation is the incomplete availability of PHQ-9 data. Although baseline comparisons did not show differences in our primary exposure variables, missing symptom data may still introduce uncertainty in estimating depression prevalence and its longitudinal associations. Accordingly, our findings should be interpreted with appropriate caution, and future studies with more complete patient-reported outcome capture are warranted. The small proportion of surgical patients (18.3%) limits statistical power to evaluate surgical effects on depression and inflammatory markers, and attrition over 12 months (48.8%) may have influenced long-term estimates. The observational design precludes establishing causal relationships between inflammation, treatment modalities, and psychological outcomes. Subgroup differences, such as the higher stage IV disease proportion in ICC (61.9%) versus ECC (25.4%), may have influenced depression prevalence. Future prospective studies with larger, balanced cohorts are needed to validate these findings and explore targeted interventions for high-risk patients.

## Conclusion

This study demonstrates that inflammatory markers, particularly CRP, are associated with depression in CCA patients. Surgical resection was associated with a reduced risk of depression, whereas radiotherapy and hypoalbuminemia in ECC patients were associated with an increased risk of depression. Routine CRP monitoring and timely psychiatric and nutritional support may improve clinical outcomes. Future research should clarify mechanisms that link inflammation and depression in CCA.

## Supplementary Information

Below is the link to the electronic supplementary material.

Supplementary Material 1

## Data Availability

The data used to support the findings of this study are available from the corresponding author upon request.

## Abbreviations

CCA: Cholangiocarcinoma
COX-2: Cyclooxygenase-2
CRP: C-reactive protein
ECC: Extrahepatic cholangiocarcinoma
GLMM: Generalized linear mixed model
HADS: Hospital Anxiety and Depression Scale
ICC: Intrahepatic cholangiocarcinoma
NLR: Neutrophil-to-lymphocyte ratio
PHQ-9: Patient Health Questionnaire-9
PLR: Platelet-to-lymphocyte ratio
SD: Standard deviation
VIF: Variance inflation factor
