# Supplementary material for: Inflammation-associated depression in cholangiocarcinoma: impacts of surgical resection and radiotherapy
Source: Biopsychosoc Med. 2026 Jan 30;20:3. doi: 10.1186/s13030-026-00350-6 (PMC12896193; doi:10.1186/s13030-026-00350-6)
Supplement: Supplementary file 1 — Supplementary Material 1 [file 13030_2026_350_MOESM1_ESM.docx]

**Table S1.** Retention and dropout reasons at each time point throughout the study

|  | Participants Included | Not Yet Reached Time Point | Death | Declined to Continue | Transition to palliative care | Treatment transfer |
| --- | --- | --- | --- | --- | --- | --- |
| Baseline (M0) | 164 | 0 | 0 | 0 | 0 | 0 |
| Month 2 (M2) | 152 | 2 | 8 | 2 | 0 | 0 |
| Month 3 (M3) | 146 | 0 | 5 | 0 | 0 | 1 |
| Month 4 (M4) | 134 | 3 | 6 | 0 | 3 | 0 |
| Month 6 (M6) | 127 | 3 | 3 | 1 | 0 | 0 |
| Month 9 (M9) | 105 | 12 | 6 | 0 | 2 | 2 |
| Month 12 (M12) | 80 | 15 | 6 | 1 | 3 | 0 |

***Note.*** In this study, participants were enrolled on a rolling basis. The “Not Yet Reached Time Point” column indicates participants who were still active but had not yet reached these follow-up milestones. These individuals are not classified as dropouts; instead, they remain part of the ongoing cohort and will contribute data as they reach their respective follow-up milestones.

**Table S2.** Baseline demographic and clinical characteristics of patients who completed vs. did not complete the PHQ-9

|  | PHQ-9 Completed | | PHQ-9  Not Completed | | Statistic | | | |
| --- | --- | --- | --- | --- | --- | --- | --- | --- |
|  | Mean | ±SD | Mean | ±SD | χ2/ Mann-Whitney U | p | t | p |
| Sex (Male/Female) | 51 | /33 | 42 | /38 | 1.13 | 0.289 |  |  |
| Stage (I, II, III/IV) | 29 | /50 | 43 | /27 | 9.08 | 0.003 |  |  |
| Chemotherapy (yes/no) | 70 | /14 | 65 | /15 | 0.12 | 0.727 |  |  |
| Surgery (yes/no) | 9 | /75 | 21 | /59 | 6.617 | 0.010 |  |  |
| Radiation therapy (yes/no) | 5 | /79 | 10 | /70 | 2.11 | 0.146 |  |  |
| Age | 63.33 | ±12.23 | 67.93 | ±10.60 | 1811.50 | 0.022 | -2.35 | 0.020 |
| Albumin | 3.70 | ±0.70 | 3.58 | ±0.80 | 1607.50 | 0.449 | 0.92 | 0.360 |
| Natural log C-reactive protein | 3.06 | ±1.26 | 2.97 | ±1.30 | 1268.00 | 0.719 | 0.36 | 0.724 |
| Neutrophil-to-lymphocyte ratio | 5.92 | ±5.46 | 9.85 | ±23.68 | 1691.00 | 0.299 | -1.20 | 0.233 |
| Platelet-to-lymphocyte ratio | 213.64 | ±105.07 | 261.66 | ±284.33 | 1802.00 | 0.631 | -1.30 | 0.197 |
